# Comparative Evaluation of Video Recording, Tangential, and Room Light Photography in Acne Scar Severity Assessment

**DOI:** 10.1111/jocd.71161

**Published:** 2026-09-09

**Authors:** Esra Duzdemir, Eda Karaismailoglu, Gulsen Akoglu

**Affiliations:** ^1^ Department of Dermatovenereology University of Health Sciences, Gülhane Training and Research Hospital Ankara Türkiye; ^2^ Department of Medical Informatics University of Health Sciences, Gulhane School of Medicine Ankara Türkiye

**Keywords:** acne, evaluation study, light, photography, scar

## Abstract

**Background:**

Accurate assessment of acne scarring is essential for diagnosis, treatment planning, and outcome evaluation.

**Objective:**

To compare the Global Skin Assessment (GSA), Physician's Assessment of Skin Surface Roughness (PSSR), and Échelle d'évaluation clinique des cicatrices d'acné (ECCA) scores of patients' images recorded by room light photography (RLP), tangential light photography (TLP), and video recording (VR) with dermatological examination (DE) which was accepted as the reference standard method for further analysis.

**Methods:**

Medical and imaging records of 27 patients (48.1% female; mean age 25.2 ± 5.3 years) were reviewed. Agreement for GSA and PSSR was analyzed using weighted kappa coefficients. Intraclass correlation coefficients (ICC) were calculated for continuous ECCA scores. Bland–Altman analyses were performed to evaluate systematic bias and limits of agreement.

**Results:**

For GSA, RLP demonstrated weak‐to‐moderate agreement (κ ≈ 0.59), TLP showed strong agreement (κ ≈ 0.88–0.91), and VR achieved almost perfect agreement (κ up to 1.000) with DE. Similar findings were observed for PSSR, with minimal agreement for RLP (κ = 0.285) and strong to almost perfect agreement for TLP (κ = 0.863) and VR (κ = 0.965). ICC values for ECCA scores indicated poor agreement for RLP (0.365), good agreement for TLP (0.852), and excellent agreement for VR (0.975). Bland–Altman analysis revealed the lowest bias and narrowest limits of agreement for VR (−2.96), compared with TLP (−12.59) and RLP (−50.19).

**Conclusion:**

For patients with acne scars, obtaining video recordings to capture images that most closely resemble DE findings would be the most appropriate approach before treatment and during follow‐up. If video recording is not feasible, photographing under tangential light should be preferred as an alternative.

## 1 Introduction

Acne scars are a frequent and distressing complication of acne vulgaris. They arise from abnormal wound healing responses following cutaneous inflammation, resulting in atrophic, hypertrophic, or keloid scars. Among these, atrophic scars are the most common and are classified as ice pick, rolling, and boxcar types [1].

Although various treatment options have been proposed, there is no universally accepted standard approach for acne scars [2, 3]. Therefore, the management of acne scars remains challenging and may require prolonged treatment and follow‐up. Objective evaluation of scars before treatment and at regular intervals during follow‐up is essential for accurately assessing treatment response and guiding clinical decisions.

Clinical photography plays a central role in the documentation and follow‐up of acne scars. Standardized images allow comparison over time and provide valuable support for clinical assessment. However, studies focusing on optimal imaging techniques for acne scars are limited. Estrella Porter et al. emphasized that tangential lighting is necessary to adequately visualize scar depth and volume in photographic assessments [4]. Despite this, different imaging methods are still used in routine practice, and their relative value has not been clearly established.

In the present study, we compared dermatological examination (DE), photography under room light, photography under tangential lighting, and video recordings to evaluate whether these methods differ in their assessment of acne scar severity and skin appearance scores.

## 2 Materials and Methods

The study population consisted of 27 patients who were followed in our clinic for acne scars. Medical records and digital images of the patients were reviewed retrospectively, and their demographic and clinical characteristics were recorded.

The Échelle d'évaluation clinique des cicatrices d'acné (ECCA) grading scores, as well as the Global Skin Assessment (GSA) and Physician's Assessment of Skin Surface Roughness (PSSR) scores documented during DE, were obtained from patient records. In addition, photographs taken under room light and tangential lighting, and video recordings obtained at the same visit were reviewed, and the same scores were evaluated from these images.

### 2.1 Measurements

The following scores were assessed and recorded for each patient during DE, from photographs, and from video recordings. All measurements were performed by the same dermatologist during DE in the same examination room with standard ceiling room lighting and DE was accepted as the reference standard method for further analysis. The same measurements were scored for the evaluation of the photographs and video recordings by the same dermatologist. GSA was scored separately for the right and left sides of the face, whereas PSSR and ECCA were each recorded as a single overall score for the entire face.

#### 2.1.1 Global Skin Assessment

The severity of acne scars was evaluated based on the surface area involved on one half of the face. The extent of scarring was graded on a scale from 0 to 4 (0 = no post‐acne lesions; 1 = involvement of less than 25% of one half of the face; 2 = involvement of 25%–50%; 3 = involvement of 50%–75%; and 4 = involvement of more than 75%).

#### 2.1.2 Physician's Assessment of Skin Surface Roughness

The severity of acne scars was evaluated according to the degree of skin surface roughness. Roughness was graded visually on a scale from 0 to 4 (0 = smooth skin; 1 = almost smooth; 2 = mildly rough; 3 = moderately rough; and 4 = severely rough).

#### 2.1.3 ECCA Grading Scale

The ECCA grading scale, described by Dreno et al., was developed as a tool for use in clinical practice and in clinical studies evaluating treatment efficacy in acne scars [5]. The scale consists of six items corresponding to six types of acne scars, defined according to their qualitative characteristics. Each scar type is assigned a semi‐quantitative score (ranging from 0 to 3) and a weighting factor (ranging from 15 to 50).

The semi‐quantitative score reflects the number of scars (0 = no scars; 1 = few scars [< 5]; 2 = limited number of scars [5–20]; 3 = numerous scars [> 20]). The weighting factor reflects the morphological features of the scar: 15 for atrophic V‐shaped scars (diameter < 2 mm, punctiform), 20 for atrophic U‐shaped scars (diameter 2–4 mm, sharply demarcated), 25 for atrophic M‐shaped scars (diameter > 4 mm, superficial with irregular margins), 30 for superficial elastolysis, 40 for hypertrophic inflammatory scars (< 2 years duration), and 50 for keloid or hypertrophic scars (> 2 years duration).

For each scar type, the semi‐quantitative score is multiplied by the corresponding weighting factor, and the total ECCA score is calculated as the sum of these values. The total score provides a quantitative measure of acne scar severity.

### 2.2 Photography and Video Recording

All imaging procedures, photography and video recordings, were performed by the same dermatologist after DE in the same examination room with standard ceiling room lighting. Before both DE and imaging procedures, all cosmetic products (including sunscreens) and topical treatments were removed from the patients' facial skin using an appropriate skin cleanser. In male patients with facial hair, evaluation and imaging were performed after shaving.

Room light photography (RLP) was performed in the same room, under identical ceiling lighting conditions, with the patient positioned in front of the same wall where a dark blue background was mounted. For tangential light photography (TLP), the patient was positioned in front of the same wall, and the smartphone light source was directed toward the patient's face at an angle of approximately 15–20 degrees [6]. For video recording (VR), the patient was again positioned in front of the same wall under tangential lighting, and the entire face was recorded.

All photographs and videos were captured using the same smartphone camera (Samsung Electronics Galaxy S23). Photographs were taken at a resolution of 12 megapixels (3000 × 4000 pixels). Video recordings were obtained at 8 K resolution (7680 × 4320 pixels) with a frame rate of 30 frames per second. To ensure consistency, all images and videos were acquired under similar conditions with respect to lighting, camera angle, patient positioning, and shooting distance at each visit. The images and video recordings were digitally stored without any modification to image quality or settings.

### 2.3 Ethics

The study was approved by the local ethics committee (approval date and no: March 6, 2025; 2025/43). Written informed consent was obtained from all patients prior to clinical photography and video recording.

### 2.4 Statistical Analysis

All statistical analyses were performed using SPSS Statistics version 23 (IBM, Chicago, IL, USA) and R statistical software (version 4.3.0; The R Foundation for Statistical Computing). Numerical variables were presented as the mean ± standard deviation and median (minimum–maximum). The agreement between the three methods and DE (the reference method) for the ordinal variables was assessed using the weighted kappa coefficient. A quadratic weighting technique was utilized to account for the magnitude of disagreement. Kappa values were interpreted as follows: values between 0.00–0.20 indicate no agreement, 0.21–0.39 indicate minimal agreement, 0.40–0.59 indicate weak agreement, 0.60–0.79 indicate moderate agreement, 0.80–0.90 indicate strong agreement, and values above 0.90 indicate almost perfect agreement [7]. The intraclass correlation coefficient (ICC) was used for continuous variables. Bland–Altman plots were used to visually inspect systematic bias and the dispersion of differences across the range of measurements for continuous ECCA scores. The mean difference (bias) and the 95% limits of agreement (LoA) (bias ±1.96 × SD_Difference_) were calculated. A two‐sided *p* value of less than 0.05 was considered statistically significant.

## 3 Results

**FIGURE 1:**
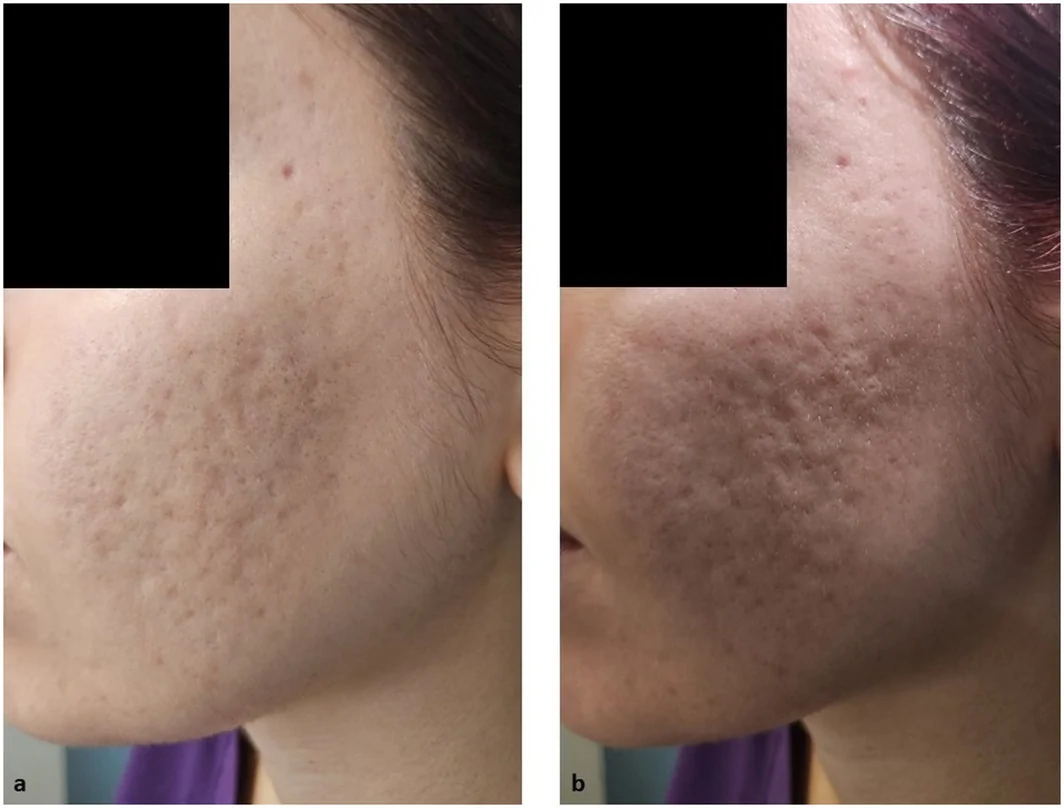
Representative photographs of acne scars in a patient taken under (a) room light and (b) tangential lighting. The GSA, PSSR, and ECCA scores from the room light photograph were 2, 2, and 110, respectively, and from the tangential light photograph were 3, 3, and 130, respectively.

Of the 27 patients, 13 (48.1%) were female and 14 (51.9%) were male. The patients' ages ranged from 18 to 39 years, with a mean age of 25.2 ± 5.3 years. Tables 1 and 2 demonstrate the scores of GSA, PSSR, and ECCA scores evaluated by DE and by analysis of photographs obtained under room light and tangential lighting, together with video recordings of the patients. Figure 1; Figure S1, and Video S1 show representative images of two patients.

**TABLE 1:** Global skin assessment, physician's assessment of skin surface roughness, and ECCA scores evaluated by dermatological examination and by analysis of photographs obtained under room light and tangential lighting, together with video recordings of the patients.

Patient no	Global skin assessment‐right face				Global skin assessment‐left face				Physician's assessment of skin surface roughness				ECCA			
	RLP	TLP	VR	DE	RLP	TLP	VR	DE	RLP	TLP	VR	DE	RLP	TLP	VR	DE
1	2	3	4	4	3	4	4	4	2	4	4	4	130	145	160	180
2	3	4	4	4	3	3	4	4	2	3	4	4	130	165	165	180
3	1	2	2	2	1	1	2	2	2	3	3	3	110	130	130	130
4	2	3	3	3	2	3	3	3	2	3	3	3	90	145	145	145
5	3	4	4	4	2	3	3	3	2	4	4	4	140	165	180	180
6	1	2	2	2	1	2	2	2	1	2	3	3	75	155	155	155
7	2	3	3	3	2	3	3	3	2	3	3	3	105	135	165	165
8	3	4	4	4	3	4	4	4	3	4	4	4	110	150	150	165
9	4	4	4	4	3	4	4	4	3	4	4	4	195	250	270	270
10	4	4	4	4	4	4	4	4	3	4	4	4	150	150	180	180
11	1	2	2	2	1	2	2	2	2	3	3	3	110	135	135	135
12	2	2	2	2	2	3	3	3	2	3	3	3	110	130	145	145
13	1	2	2	2	1	2	2	2	2	3	3	3	70	110	130	145
14	3	3	3	3	2	3	3	3	2	4	4	4	85	165	180	180
15	2	3	3	3	2	3	3	3	2	3	3	3	80	150	165	165
16	3	3	3	3	3	3	3	3	3	3	4	4	135	165	165	180
17	2	3	3	3	2	3	3	3	2	3	3	4	85	130	130	130
18	3	4	4	4	3	4	4	4	3	4	4	4	150	165	180	180
19	1	1	2	2	1	1	2	2	1	2	2	2	70	135	135	135
20	3	3	3	4	2	3	3	3	3	4	4	4	130	150	165	165
21	2	3	3	3	2	3	3	3	2	3	3	3	120	165	180	180
22	2	3	3	3	2	3	3	3	2	3	3	3	115	160	160	160
23	1	1	2	2	1	1	2	2	2	2	2	2	95	115	135	135
24	2	2	2	2	1	2	2	2	2	2	2	2	75	115	135	135
25	1	2	2	2	1	2	2	2	1	2	2	2	100	135	135	135
26	2	3	3	3	2	2	2	2	2	3	3	3	180	195	195	195
27	1	1	2	2	1	1	1	1	1	2	2	2	60	110	110	110

Abbreviations: DE, dermatological examination; RLP, room light photography; TLP, tangential lighting photography; VR, video recordings.

**TABLE 2:** Descriptive statistics of global skin assessment, physician's assessment of skin surface roughness, and ECCA scores evaluated using four methods.

Variable	Method	Mean ± standard deviation	Median (minimum–maximum)
Global skin assessment: right face	RLP	2.11 ± 0.93	2 (1–4)
	TLP	2.74 ± 0.94	3 (1–4)
	VR	2.89 ± 0.80	3 (2–4)
	DE	2.93 ± 0.83	3 (2–4)
Global skin assessment: left face	RLP	1.96 ± 0.85	2 (1–4)
	TLP	2.67 ± 0.96	3 (1–4)
	VR	2.81 ± 0.83	3 (1–4)
	DE	2.81 ± 0.83	3 (1–4)
Physician's assessment of skin surface roughness	RLP	2.07 ± 0.62	2 (1–4)
	TLP	3.07 ± 0.73	3 (1–4)
	VR	3.18 ± 0.74	3 (2–4)
	DE	3.22 ± 0.75	3 (1–4)
ECCA scores	RLP	111.29 ± 33.50	110 (60–195)
	TLP	148.89 ± 28.60	150 (110–250)
	VR	158.52 ± 30.57	160 (110–270)
	DE	161.48 ± 30.82	165 (110–270)

Abbreviations: DE, dermatological examination; RLP, room light photography; TLP, tangential lighting photography; VR, video recordings.

Weighted kappa coefficients demonstrated substantial differences in agreement between methods (Table 3). For “GSA‐right face,” agreement with DE was weak for RLP (κ = 0.589, 95% CI: 0.460–0.718), strong for TLP (κ = 0.881, 95% CI: 0.802–0.960), and almost perfect for VR (κ = 0.971, 95% CI: 0.915–1.000). For “GSA‐left face,” RLP demonstrated moderate agreement (κ = 0.594, 95% CI: 0.483–0.704), whereas both TLP (κ = 0.906, 95% CI: 0.830–0983) and VR (κ = 1.000, 95% CI: 1.000–1.000) exhibited almost perfect agreement with DE. Similarly, for “PSSR,” RLP showed minimal agreement (κ = 0.285, 95% CI: 0.140–0.430), while TLP (κ = 0.863, 95% CI: 0.729–0.996) and VR (κ = 0.965, 95% CI: 0.896–1.000) demonstrated strong to almost perfect agreement with the reference standard, DE.

**TABLE 3:** Weighted kappa coefficients comparing each method with dermatological examination as the reference standard.

Variable	Method	Weighted kappa	95% CI	*p*	Agreement
Global skin assessment: right face	RLP	0.589	0.460–0.718	< 0.001	Weak
	TLP	0.881	0.802–0.960	< 0.001	Strong
	VR	0.971	0.915–1.000	< 0.001	Perfect
Global skin assessment: left face	RLP	0.594	0.483–0.704	< 0.001	Moderate
	TLP	0.906	0.830–0.983	< 0.001	Perfect
	VR	1.000	1.000–1.000	< 0.001	Perfect
Physician's assessment of skin surface roughness	RLP	0.285	0.140–0.430	< 0.001	Minimal
	TLP	0.863	0.729–0.996	< 0.001	Strong
	VR	0.965	0.896–1.000	< 0.001	Perfect

Abbreviations: DE, dermatological examination; RLP, room light photography; TLP, tangential lighting photography; VR, video recordings.

Intraclass correlation analyses performed for the continuous ECCA scores reinforced these findings. Agreement with DE was poor for RLP (ICC = 0.365, 95% CI: −0.065 to 0.742), good for TLP (ICC = 0.852, 95% CI: 0.235–0.954), and excellent for VR (ICC = 0.975, 95% CI: 0.937–0.989). Bland–Altman plots further illustrated these patterns, showing wide limits of agreement and systematic bias for RLP, narrower limits and reduced bias for TLP, and the closest correspondence to the DE measurements for VR.

**FIGURE 2:**
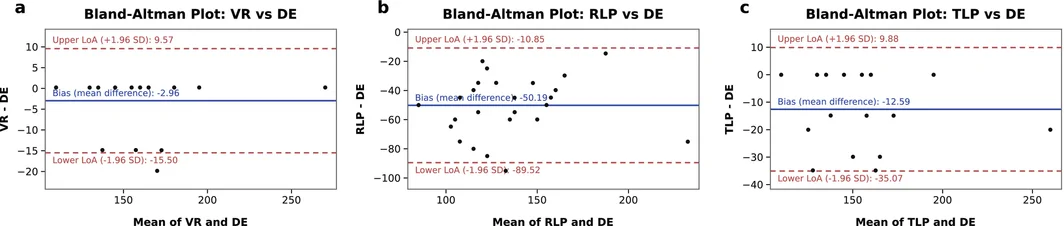
The Bland Altman plot that displays the agreement (a) between VR and DE measurements; (b) between RLP and DE measurements and (c) between TLP and DE measurements.

Bland–Altman analysis demonstrated that the VR method exhibited the strongest agreement with the DE, evidenced by the lowest mean difference (bias = −2.96) and the narrowest 95% limits of agreement (Figure 2a). This high precision confirms the potential interchangeability of the VR with DE. Conversely, RLP showed the weakest performance, marked by a large negative systematic error (bias = −50.19) and an extremely wide limits of agreement suggesting it is not a feasible alternative for DE (Figure 2b). TLP fell between the two alternative approaches, demonstrating a slightly greater systematic underestimation (bias = −12.59) and a wider limit of agreement compared to the VR (Figure 2c).

A post hoc power analysis was performed to evaluate the reliability of the measurements using G*Power software (version 3.1.9.7). The study achieved a statistical power of 95.3% to detect an ICC of 0.852, assuming a null hypothesis of 0.50 at a significance level of 0.05 with a sample size of 27.

## 4 Discussion

Our study showed that photographs and video recordings obtained under tangential illumination, whether taking photos or recording videos, significantly enhanced the visibility of atrophic scar areas. Given its low cost and ease of application, this method can be readily incorporated into routine dermatology practice for the follow‐up and documentation of patients with acne scars. Additionally, storing photographic and video records allows retrospective comparison of scar changes over time when needed in the follow‐up of patients.

In dermatology practice, obtaining reliable and standardized clinical photographs is essential for accurate documentation, patient management, long‐term follow‐up of chronic conditions, and educational purposes [8]. In the assessment of acne severity, an ideal photographic grading system should be both accurate and reproducible, while also being practical in terms of time, ease of application, and cost. However, photography remains a two‐dimensional modality and does not permit palpation, which limits the evaluation of lesion depth. Hereby, this limitation is considered one of its major drawbacks [9].

Clinical photographs of acne scars should ideally deliver information on scar type, depth, and overall skin surface irregularity during follow‐up. In one study, a dedicated digital imaging system combined with image‐analysis software was used to define and evaluate three‐dimensional (3D) parameters for grading atrophic acne scars. The authors reported that 3D imaging provided results comparable to clinical examination and demonstrated good reproducibility [10]. Nevertheless, the routine use of 3D imaging systems remains limited, largely because of their cost and restricted availability. For this reason, their use in daily practice and in all patients may not be feasible under real‐world conditions.

In the present study, tangential lighting was used as a direct illumination technique in which the light source is directed toward the skin surface at a very low angle. This approach does not require specialized imaging systems. During image acquisition, a simple flashlight or the light source of a mobile device can be used. The distance between the light source and the skin is generally maintained at approximately 5–10 cm and may be adjusted according to the characteristics of the device used [11].

Tangential lighting has also been employed in various areas of dermatology, including cellulite photography, improving the visualization of leukotrichia in patients with vitiligo, identifying biopsy sites, and examining hypopigmented dysplastic melanocytic nevi [12, 13, 14, 15]. In these studies, investigators generally reported that tangential illumination provided better visualization than standard room lighting during the examination, identification, and documentation of different dermatologic conditions. Accordingly, the routine use of tangential light has been recommended to facilitate a more accurate and rapid assessment of skin and hair lesions [11].

### 4.1 Limitations

This study has several limitations. First, the amount of sunlight entering the room during imaging could not be fully standardized and may have varied between visits to some extent, even for the same patient. This variation might have influenced the acne scar severity scores obtained from the photographs. Second, although tangential lighting was used for both photography and video recordings, the smartphone light source was handheld and not fixed in position. Therefore, slight differences in the angle and intensity of illumination may have occurred, which could have affected the visibility of scar features and the corresponding severity scores [16]. Another limitation is that all evaluations were performed by a single dermatologist. Although this ensured consistency, interobserver variability could not be assessed. The retrospective design may have limited full standardization of imaging conditions. Finally, the relatively small sample size and single‐center design may limit the generalizability of the findings.

Despite these limitations, all assessments were performed in the same room and under consistent lighting conditions, which helped to ensure uniform imaging conditions. In addition, although the sample size was relatively small, the calculated statistical power was high, supporting the reliability of the findings. The standardized evaluation of each patient using multiple assessment methods also represents an important strength of the study. Overall, the findings of our study show that photographs and video recordings obtained under tangential lighting may serve as a practical alternative, especially in centers where 3D imaging systems are not available or their routine use is impractical. This method appears to show a high level of agreement with DE in the assessment of acne scars and can be readily integrated into everyday clinical practice.

## 5 Conclusion

Standardized imaging of acne scars before treatment and at regular intervals during follow‐up contributes to a more objective evaluation of treatment response. Video recording appears to be particularly useful for assessing scar type and severity and is recommended when feasible. When video recording is not available, photography under tangential lighting may be preferred.

## Author Contributions

Esra Duzdemir contributed to data curation, formal analysis, investigation, methodology, and writing. Eda Karaismailoglu contributed to data curation, formal analysis, statistical analyses, methodology, and writing. Gulsen Akoglu contributed to the conceptualisation, formal analysis, methodology, supervision, and writing. All authors read and approved the final version of the manuscript and take full responsibility for the integrity and accuracy of all aspects of the work.

## Funding

The authors have nothing to report.

## Disclosure

No editing services were involved and no AI tools were used in the preparation of the manuscript.

## Ethics Statement

The study was approved by the local ethics committee.

## Consent

We confirm that written consent for permission to publish was obtained from participants to publish any recognizable images of people.

## Conflicts of Interest

The authors declare no conflicts of interest.

## Supporting information


**Figure S1:** Representative photographs of acne scars in a patient taken under (a) room light and (b) tangential lighting. The GSA, PSSR, and ECCA scores from the room light photograph were 3, 2, and 130, respectively, and from the tangential light photograph were 4, 4, and 145, respectively.
**Video S1:** Video recording of the acne scars of the same patient. The GSA, PSSR, and ECCA scores from the video recording were 4, 4, and 160, respectively, and from the dermatological examination (reference method) were 4, 4, and 180, respectively.

## Data Availability

The data that support the findings of this study are available from the corresponding author upon reasonable request.
